# Supplementary material for: Comparison of Oncologic Outcomes between Transduodenal Ampullectomy and Pancreatoduodenectomy in Ampulla of Vater Cancer: Korean Multicenter Study
Source: Cancers (Basel). 2021 Apr 23;13(9):2038. doi: 10.3390/cancers13092038 (PMC8122928; doi:10.3390/cancers13092038)
Supplement: Supplementary file 1 [file cancers-13-02038-s001.zip › cancers-1194712-supplementary.pdf]

Table S1. Comparison of clinicopathologic characteristics and recurrence between the PPPD and TDA groups in the propensity score matched analysis.

	Tis, T1, and T2			Tis and T1			T1		
	**PPPD (68)**	**TDA (68)**	**p-Value**	**PPPD (55)**	**TDA (55)**	**p-Value**	**PPPD (31)**	**TDA (31)**	**p-Value**
Age (years)	62.0 ± 8.3	61.2 ± 12.7	0.649	63.3 ± 9.6	60.1 ± 13.0	0.140	63.6 ± 11.0	60.6 ± 14.3	0.348
Male (%)	37 (54.4%)	36 (52.9%)	0.863	32 (58.2%)	28 (50.9%)	0.444	16 (51.6%)	15 (48.4%)	0.799
Size (cm)	1.9 ± 1.0	1.6 ± 0.8	0.085	1.9 ± 0.9	1.6 ± 0.8	0.128	1.8 ± 1.1	1.4 ±0.8	0.121
T staging			<0.001			<0.001			
Tis	5 (7.4%)	24 (35.3%)		5 (9.1%)	24 (43.6%)				
T1	52 (76.5%)	31 (45.6%)		50 (90.9%)	31 (56.4%)				
T2	11 (16.2%)	13 (19.1%)							
N staging			<0.001			<0.001			0.169
Nx	8 (11.8%)	42 (61.8%)		5 (9.1%)	35 (63.6%)		5 (16.1%)	18 (58.1%)	
N0	59 (86.8%)	24 (35.3%)		46 (83.6%)	19 (34.5%)		22 (71.0%)	12 (38.7%)	
N1	1 (1.5%)	2 (2.9%)		4 (7.3%)	1 (1.8%)		4 (12.9%)	1 (3.2%)	
Differentiation			0.130			0.048			0.820
Well	46 (67.6%)	28 (41.2%)		42 (76.4%)	24 (43.6%)		20 (64.5%)	22 (71.0%)	
Moderate	8 (11.8%)	8 (11.8%)		4 (7.3%)	4 (7.3%)		4 (12.9%)	4 (12.9%)	
Poorly	1 (1.5%)	1 (1.5%)		0 (0.0%)	0 (0.0%)		0 (0.0%)	0 (0.0%)	
Undiff.	0 (0.0%)	0 (0.0%)		0 (0.0%)	0 (0.0%)		0 (0.0%)	0 (0.0%)	
Etc.	5 (7.4%)	14 (20.6%)		3 (5.5%)	13 (23.6%)		2 (6.5%)	2 (6.5%)	
LVI (+)	0 (0.0%)	0 (0.0%)		0 (0.0%)	0 (0.0%)		0 (0.0%)	0 (0.0%)	
PNI (+)	2 (2.9%)	1 (1.5%)	1.000	0 (0.0%)	0 (0.0%)		0 (0.0%)	0 (0.0%)	
Adj Tx	12 (17.6%)	7 (10.3%)	0.216	4 (7.3%)	1 (1.8%)	0.363	4 (12.9%)	1 (3.2%)	0.354
R status			0.115			0.243			0.492
R0	67 (98.5%)	62 (91.2%)		55 (51.4%)	52 (94.5%)		31 (100.0%)	29 (93.5%)	
R1	1 (1.5%)	6 (8.8%)		0 (0.0%)	3 (5.5%)		0 (0.0%)	2 (6.5%)	
Recurrence	9 (13.2%)	11 (16.2%)	0.628	5 (9.1%	8 (14.5%)	0.376	3 (9.7%)	8 (25.8%)	0.096
Recurrence pattern			0.092			0.565			0.236
Local	2 (2.9%)	7 (10.3%)		1 (1.8%)	4 (7.3%)		0 (0.0%)	4 (12.9%)	
Systemic	7 (10.3%)	4 (5.9%)		4 (7.3%)	4 (7.3%)		3 (9.7%)	4 (12.9%)	

PPPD: pylorus preserving pancreaticoduodenectomy, TDA: transduodenal ampullectomy, Tis: carcinoma in situ, Undiff.: undifferentiated, LVI: lymphovascular invasion, PNI: perineural invasion, adj. Tx: adjuvant treatment.
